# Baseline soluble MICA levels act as a predictive biomarker for the efficacy of regorafenib treatment in colorectal cancer

**DOI:** 10.1186/s12885-022-09512-5

**Published:** 2022-04-20

**Authors:** Jun Arai, Yumi Otoyama, Ken-ichi Fujita, Kaku Goto, Masayuki Tojo, Atsushi Katagiri, Hisako Nozawa, Yutaro Kubota, Takehiro Takahashi, Hiroo Ishida, Takuya Tsunoda, Natsumi Matsumoto, Keita Ogawa, Ryo Nakagawa, Ryosuke Muroyama, Naoya Kato, Hitoshi Yoshida

**Affiliations:** 1 Division of Gastroenterology, Department of Medicine, Showa University School of Medicine, 1-5-8 Hatanodai, Shinagawa-ku, Tokyo, Japan; 2 Division of Cancer Genome and Pharmacotherapy, Department of Clinical Pharmacy, Showa University School of Pharmacy, Tokyo, Japan; 3 Institut de Recherche Sur Les Maladies Virales Et Hépatiques, INSERM, Strasbourg, France; 4 Division of Medical Oncology, Department of Medicine, Showa University School of Medicine, Tokyo, Japan; 5 Division of Medical Oncology, Showa University Fujigaoka Hospital, Yokohama, Japan; 6 Division of Internal Medicine, Department of Medicine, Showa University Hokubu Hospital, Yokohama, Japan; 7 Department of Gastroenterology, Graduate School of Medicine, Chiba University, Chiba, Japan

**Keywords:** Colorectal Cancer, Regorafenib, MHC class I polypeptide-related sequence A, Natural killer cell

## Abstract

**Background:**

To evaluate the effect of regorafenib on soluble MHC class I polypeptide-related sequence A (MICA) (sMICA) level in vitro. In addition, we clinically examined whether its plasma levels were associated with regorafenib activity in terms of progression-free survival (PFS) in patients with CRC.

**Methods:**

Human CRC cell line HCT116 and HT29 cells were treated with regorafenib and its pharmacologically active metabolites, M2 or M5 at the same concentrations as those in sera of patients. We also examined the sMICA levels and the area under the plasma concentration–time curve of regorafenib, M2 and M5.

**Results:**

Regorafenib, M2, and M5 significantly suppressed shedding of MICA in human CRC cells without toxicity. This resulted in the reduced production of sMICA. In the clinical examination, patients with CRC who showed long median PFS (3.7 months) had significantly lower sMICA levels than those with shorter median PFS (1.2 months) (*p* = 0.045).

**Conclusions:**

MICA is an attractive agent for manipulating the immunological control of CRC and baseline sMICA levels could be a predictive biomarker for the efficacy of regorafenib treatment.

## Background

Recent progress in the field of cancer immunology has provided a better understanding of cancer microenvironments in which tumor cells and immune cells interact with each other. Natural killer (NK) cells are large granular lymphocytes with natural cytotoxicity against tumor cells [1]. NK cells express several receptors on the cell surface that are formed by noncovalent interactions between distinct transmembrane ligand-binding and signaling adaptor polypeptides [2]. In particular, the activity of membrane-bound MHC class I polypeptide-related sequence A (MICA) (mMICA), which is expressed on the surface of cancer cells, in turn activates cytolytic responses of NK cells against epithelial tumor cells [3]. 

To improve therapeutic efficacy in activating NK cells via mMICA, many studies have focused on hepatocellular carcinoma (HCC). In Japan, hepatitis B or C viral infection is a major cause of HCC [4]. In our previous genome-wide association study, *MICA* was identified as a susceptible gene for HCC induced by hepatitis B or C virus [5]. We clarified that the risk allele of A at rs2596542 in *MICA* was associated with lower soluble MICA (sMICA) protein levels in plasma in individuals with hepatitis C virus-induced HCC compared with the non-risk G allele. mMICA, which is expressed on the surface of tumor cells, is prone to proteolytic cleavage to release sMICA, which then acts as an immunological decoy in the serum to prevent immune-related anti-tumor activity by immune cells such as NK cells [6]. Our recent study clarified that several A disintegrin and metalloproteases (ADAMs), including ADAM9, are MICA sheddases that are involved in HCC and that the suppression of ADAMs demonstrates the rationality to enhance MICA-NK-targeted therapy [7]. Therefore, high plasma levels of sMICA have been shown to be an indicator of poor prognosis in patients with HCC induced by chronic hepatitis B or hepatitis C [8]. In cohorts of patients with chronic hepatitis C, higher sMICA levels after viral eradication were associated with HCC development [9].

Regorafenib is an analog of sorafenib that is known to disrupt the tumor microenvironment and prevent angiogenesis [10–12]. In the Phase III RESORCE trial, regorafenib was shown to provide a survival advantage to patients with HCC who are resistant to sorafenib [13]. Regorafenib improved overall survival with a hazard ratio of 0.63 (median survival 10.6 months for the drug versus 7.8 months for the placebo). However, the immunological mechanism of regorafenib activity in HCC remains unclear, considering there are no baseline biomarkers to predict its efficacy [14, 15]. We have reported that regorafenib inhibits MICA shedding to a greater extent than sorafenib for suppressing the transcription of ADAM9 in human HCC cells [16], without a marked difference in cytotoxicity. Importantly, antifungal lomofungin [7], disulfiram [17], leukotriene receptor antagonists [18], and retinoids [19] are also shown to be potential drugs that enhance NK cell immunity against human HCC by suppressing ADAM enzymatic activity to decrease sMICA production in HCC cells.

MICA has also been found to be expressed in freshly isolated colorectal cancer (CRC) specimens and HCT116 human CRC cells [20]. CRC is the second leading cause of cancer-related death worldwide. Approximately 20% of patients are diagnosed with metastatic diseases (stage IV) in the first clinical examination, and approximately one-third of treated patients who received pharmacotherapy experience relapse [21]. Regorafenib is the first small-molecule tyrosine kinase inhibitor to provide survival benefits to patients with metastatic CRC with disease progression even with different standard therapies [22]. To date, the immunological features in patients with CRC treated with regorafenib have not been well clarified. Regorafenib is sequentially metabolized primarily in the liver by cytochrome P450 3A4 to form two major metabolites, regorafenib (pyridine)-*N*-oxide (M2) and *N-*desmethyl regorafenib (pyridine)-*N*-oxide (M5), both of which possess pharmacological activities similar to that of regorafenib [23].

In this study, we evaluated the effects of regorafenib and its metabolites M2 and M5 on MICA shedding in human CRC cell lines in vitro, and we clinically investigated whether sMICA levels in plasma are associated with the efficacy of regorafenib in terms of progression-free survival (PFS) in patients with CRC.

## Methods

### Cells, reagents, and antibodies

Regorafenib was obtained from Cell Signaling Technology (Danvers, MA, USA). M2 and M5 were purchased from SHIMADZU GLC Ltd. (Tokyo, Japan). The Cell Counting Kit-8 (CCK8) was purchased from Dojindo (Kumamoto, Japan). The ON-TARGETplus SMARTpool siRNA duplex mixtures of siADAM9, siADAM10, siADAM17, siADAM21, siMT1-MMP, siMMP2, and siMMP9 were purchased from Dharmacon (Lafayette, CO, USA). Non-targeting control siRNA and Isogen II reagents were obtained from Qiagen (Hilden, Germany) and Nippon Gene (Tokyo, Japan), respectively.

HCT116 and HT29 cells were obtained from the American Type Culture Collection (Manassas, VA, USA) and cultured according to the supplier’s protocols. The cell lines were authenticated by short tandem repeat analysis (Bex, Tokyo, Japan) in November 2020.

### Cell viability assays

HCT116 and HT29 cells (2 × 10^5^ cells/mL/well) were plated on 24-well plates and incubated at 37 °C for 24 h. Then, the cells were treated with regorafenib, M2, or M5 for 48 h. After the treatment, the culture supernatant was removed and cell viability was measured using the CCK8 assay kit. Briefly, CCK-8 reagent diluted following the manufacturer’s instructions was added to each well and the plates were incubated at 37 °C for 1 h. After incubation, absorbance was measured at a wavelength of 450 nm using a microplate reader to determine the number of viable cells.

### Enzyme-linked immunosorbent assay (ELISA)

sMICA levels in the HCT116 and HT29 cell culture supernatants and sera of patients were measured using a MICA ELISA Kit (Diaclone, Besançon, France) as described previously [7]. In the cell culture supernatants, relative sMICA was calculated by dividing the sMICA level by the degree of cell viability evaluated with the CCK8 assay.

### Knockdown of ADAMs and MMPs with siRNA

For the specific knockdown of ADAM9, ADAM10, ADAM17, ADAM21, MT1-MMP, MMP2, and MMP9, the ON-TARGETplus SMARTpool siRNA duplex mixtures were used as described previously [7]. The silencing efficiency and specificity of the siRNAs were checked by the supplier. Non-targeting control siRNA was used as a control for nonspecific silencing effects.

### Quantitative reverse transcription-polymerase chain reaction (qRT-PCR)

Total RNA was extracted using the Isogen II reagent according to the manufacturer’s instructions. RNA was reverse transcribed into complementary DNA with the Superscript First-Strand Synthesis System for RT-PCR kit (Invitrogen, Carlsbad, CA, USA). Relative mRNA levels were quantified as previously described [16] using the following primer sets:

MICA-F: 5′-CTTCCTGCTTCTGGCTGGCATC-3′,

MICA-R: 5′-CAGGGTCATCCTGAGGTCCTTTC-3′,

ADAM9-F: 5′-AAGAATTGTCACTGTGAAAATGGCT-3′,

ADAM9-R: 5′-CATTGTATGTAGGTCCACTGTCCAC-3′,

ADAM10-F: 5′-ACGGAACACGAGAAGCTGTG-3′,

ADAM10-R: 5′-CCGGAGAAGTCTGTGGTCTG-3′,

ADAM17-F: 5′-GTCGAGCCTGGCGGTAGAATCTTC-3′,

ADAM17-R: 5′-CTCCACCTCTCTGGGCAGCCTTC-3′,

GAPDH-F: 5′-ATGGGGAAGGTGAAGGTCG-3′,

GAPDH-R: 5′-GGGGTCATTGATGGCAACAATA-3′.

## Patients and plasma and genomic DNA collection

This study included patients with metastatic CRC from a previously reported prospective study (UMIN000013939) to examine the area under the unbound plasma concentration–time curve (AUCu) of regorafenib and its active metabolites, M2 and M5, to establish pharmacokinetic bases for individualized dosing strategies [23]. Regorafenib was administered orally at a dose of 160 mg/body once daily for weeks 1–3 of each 4-week cycle. We selected patients who discontinued the treatment due to disease progression but not due to adverse events. These patients were divided into two groups, responders and non-responders, as determined by the median PFS calculated for all selected patients. To examine plasma sMICA levels, we used plasma samples taken immediately before and 48 h after regorafenib administration, which were collected to examine pharmacokinetic properties of regorafenib, M2, and M5.

### Statistical analysis

All values presented indicate the mean and standard error of the mean unless otherwise indicated. Differences in the expression of sMICA levels between treatment and control groups were determined using paired, two-tailed Student’s *t*-test. The area under the curve (AUC) values were determined with receiver-operating characteristics (ROC) analysis. Overall survival (OS) and PFS were calculated using the Kaplan–Meier method and compared using the log-rank test. *P*-values of < 0.05 were considered statistically significant. All statistical analyses were performed using JMP ver. 15 (SAS Institute, Cary, NC, USA).

## Results

### ADAM10 and ADAM17 are involved in sMICA production in CRC cells

**Fig. 1:**
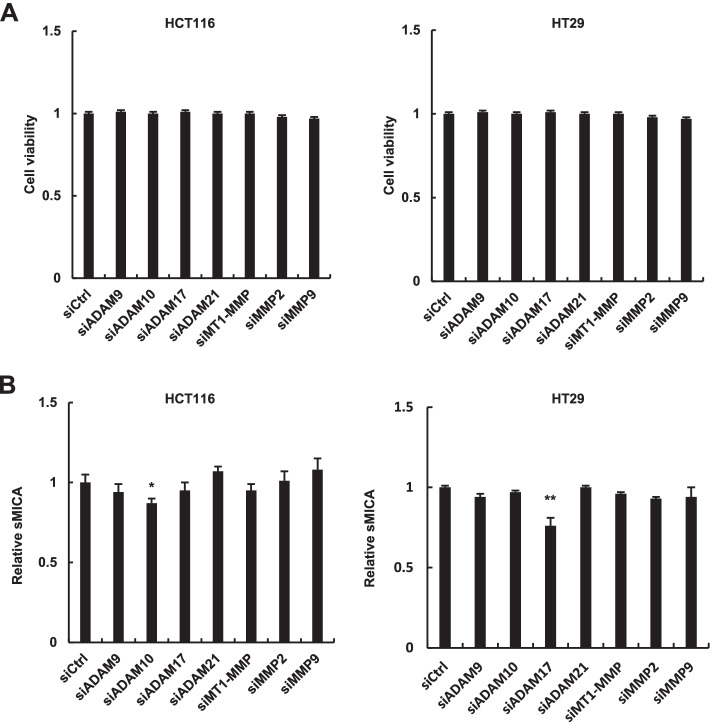
Knockdown of ADAMs and MMPs in human CRC cells. **A** HCT116 and HT29 cells were transfected with siADAM9, siADAM10, siADAM17, siADAM21, siMT1-MMP, siMMP2, siMMP9, or siCtrl; cell viabilities were determined by the CCK8 assay. **B** In HCT116 and HT29 cells, sMICA levels were determined by ELISA. *P < 0.05; **P < 0.005. Error bars represent standard error of the mean. Representative data from three independent experiments with consistently similar results are shown

First, to test the impact of ADAMs and MMPs on sMICA production, we knocked down the expression of individual genes using siRNAs by transfecting the CRC cell lines HCT116 and HT29 with siADAM9, siADAM10, siADAM17, siADAM21, siMT1-MMP, siMMP2, or siMMP9, and using siCtrl as a negative control. None of the siRNAs exerted cytotoxic effects (Fig. 1A). When measuring the supernatant of cultured cells, significant suppression of relative sMICA production was observed in HT29 cells transfected with siADAM17, whereas the relative sMICA levels in HCT116 cells was significantly decreased by siADAM10 (Fig. 1B).

## Regorafenib inhibits the release of sMICA in human CRC cells

**Fig. 2:**
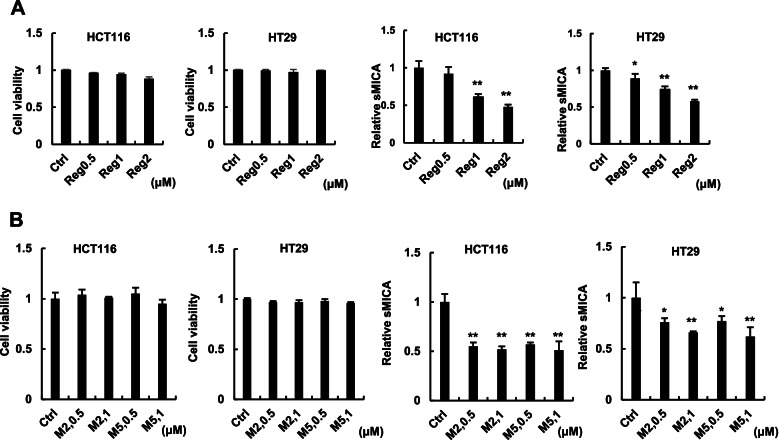
Regorafenib and its metabolites, M2 and M5, inhibited sMICA release in CRC cells. **A** HCT116 and HT29 cells were treated with regorafenib for 48 h. Cell viabilities (left panels) and sMICA levels (right panels) were determined by the CCK8 assay and ELISA, respectively. **B** HCT116 and HT29 cells were treated with M2 and M5 for 48 h. Cell viabilities (left panels) and sMICA levels (right panels) were determined by the CCK8 assay and ELISA, respectively. *P < 0.05; **P < 0.005. Error bars represent standard error of the mean. Representative data from three independent experiments with consistently similar results are shown. Reg: regorafenib

In the assessment of the cytotoxicity of regorafenib against HCT116 and HT29 CRC cells, there was no cytotoxic effect with up to 2 µM regorafenib in both cell lines (Fig. 2A). Regorafenib significantly decreased the levels of relative sMICA in both cell lines, even at a concentration of 1 µM (Fig. 2A).

## M2 and M5 also inhibit release of sMICA in human CRC cells

No cytotoxic effects were observed in HCT116 or HT29 cells treated with M2 or M5 at concentrations of 0.5 or 1 μM (Fig. 2B). M2 and M5 significantly decreased the levels of relative sMICA in both cell lines, as measured in the culture supernatant by ELISA, even at a concentration of 0.5 µM. However, regorafenib, M2, and M5 did not diminish the abundance of ADAM9, ADAM10, ADAM17, or MICA mRNA in either HCT116 or HT29 cells (data not shown).

## Patient characteristics in clinical study

Seventeen patients were selected according to the PFS as described in the Methods section. Treatment with regorafenib in these patients was discontinued due to tumor progression, but not drug-related adverse events. The median PFS in this cohort was 2.47 months. The numbers of patients categorized as responders and non-responders were eight and nine, respectively. Table 1 shows the characteristics of these participants, including the differences between responders and non-responders. All patients showed adequate liver and kidney functions, which met the eligibility criteria. Sixteen patients received three or more lines of treatment.

**Table 1:** The characteristics of participants

	*N* = 17 (%)	responders	non-responders	*P*-value
Age (years)	68 (50–77)^a^	68 (50–77)^a^	64.5 (63–74)	n.s
Gender				
Male/female	10/7 (59/41)	4/4	6/3	n.s
PS				
0/1	9/8 (53/47)	4/4	5/4	n.s
Total bilirubin (mg/dL)	0.7 (0.1–1.2)^a^	0.7(0.1–1.1)	0.7(0.4–1.2)	n.s
Serum creatinin (mg/dL)	0.69 (0.51–1.04)^a^	0.76 (0.51–1.04)	0.61 (0.51–1)	n.s
eGFR (mL/min)	80 (55.4–117.8)^a^	74.4 (55.4–89.6)	85.2 (57.1–117.8)	n.s
Histology				
Well/moderately/poor,mucinous	7/9/1 (41/53/6)	4/4/0	3/5/1	n.s
Metastatic sites				
Liver/lung/peritoneum/lymph node	11/9/2/4 (65/53/12/24)	4/7/0/1	7/2/2/2	n.s
Treatment lines				
2/3/4/5	1/9/6/1 (6/53/35/6)	0/5/2/1	1/4/4/0	n.s

Treatment lines, The number of treatment lines before regorafenib

eGFR, estimated glomerular filtration rate; PS, ECOG performance status

^a^Median (range)

## sMICA tended to be lower in responders than in non-responders

**Fig. 3:**
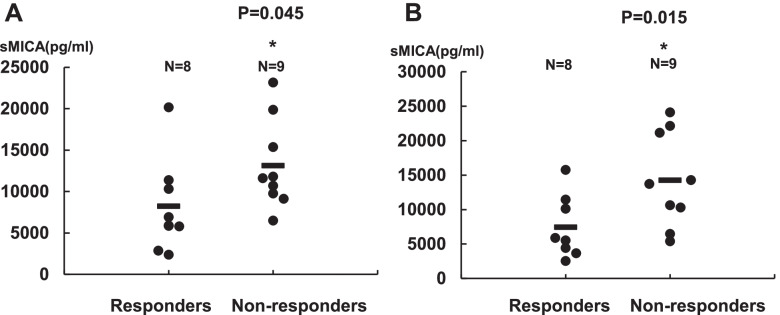
sMICA levels in CRC patients compared between responders and non-responders. **A** sMICA levels were compared between responders (*N* = 8) and non-responders (*N* = 9), before and (**B**) 48 h after regorafenib therapy. *P < 0.05. well-dif: well-differentiated

In the comparison of plasma sMICA levels measured before regorafenib treatment (baseline) between responders (*N* = 8) and non-responders (*N* = 9), responders showed significantly lower sMICA level than non-responders (*p* = 0.045) (Fig. 3A). This difference was larger after 48 h of regorafenib treatment compared with that at the pretreatment stage (*p* = 0.015) (Fig. 3B).

## ROC analysis

**Fig. 4:**
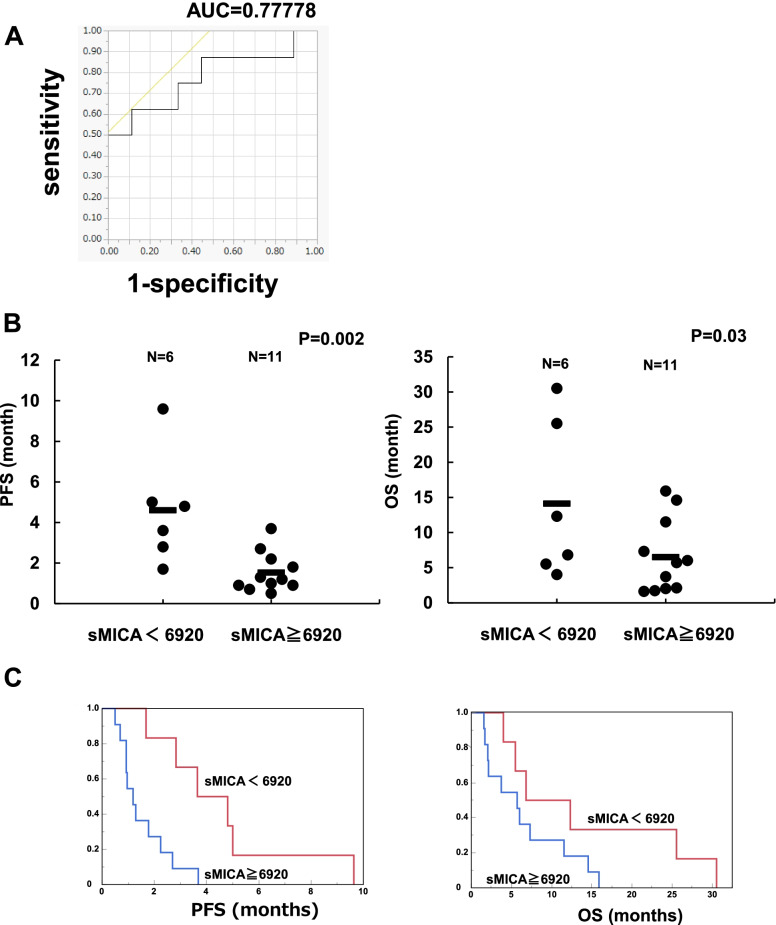
ROC analysis of sMICA and the comparison of PFS and OS according to sMICA level. **A** ROC analysis of sMICA levels for the detection of responders was conducted. **B** PFS and OS between low and high plasma sMICA level groups were analyzed. **C** Kaplan–Meier analyses of PFS and OS according to the sMICA levels were conducted. PFS: progression-free survival, OS: overall survival

Next, we conducted an ROC analysis to determine a baseline plasma sMICA level cut-off for optimally distinguishing patients in terms of response to regorafenib therapy. The AUC value for the detection of responders was 0.778 (Fig. 4A), and the optimal cut-off value of plasma sMICA levels was determined to be 6,920 ng/mL. According to this cut-off value, the patients were divided into two groups: low plasma sMICA group (*N* = 6) and high plasma sMICA group (*N* = 11).

## Effects of baseline plasma sMICA levels on the survival benefit of regorafenib

Among the 17 patients analyzed in this study, there were significant differences in PFS and OS (Fig. 4B) between the low and high plasma sMICA groups. We also analyzed the effects of the plasma sMICA levels on PFS and OS by Kaplan–Meier analyses. There was no significant difference in OS between these groups (*p* = 0.114, Fig. 4C). However, PFS was significantly shorter in the high plasma sMICA group than in the low plasma sMICA group (median PFS: 1.18 vs. 4.22 months, *p* = 0.005, Fig. 4C).

## Association of pharmacokinetics of M2 or M5 with regorafenib efficacy

**Fig. 5:**
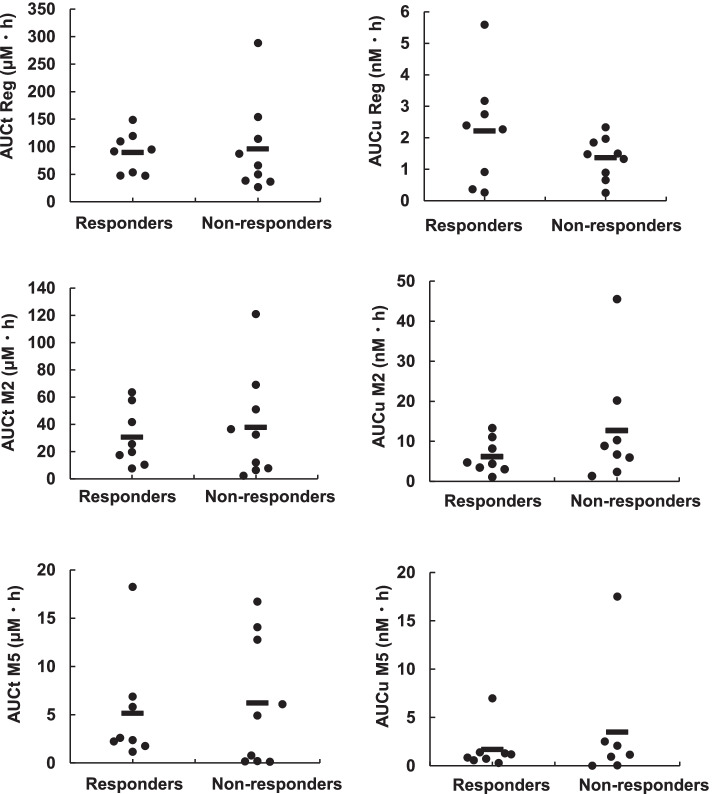
Association of pharmacokinetics of regorafenib or its active metabolites with efficacy of regorafenib. AUCt and AUCu values of regorafenib, M2, and M5 were analyzed between responders and non-responders

In our previous study [23], patients with higher AUCu values for M2 or M5 on day 1, but not total plasma concentration base AUC (AUCt) of M2 or M5, showed significantly shorter PFS than others, likely due, at least in part, to treatment discontinuation as a result of adverse events, especially during first cycle. We examined whether PFS values observed in the 17 patients who did not discontinue the regorafenib treatment due to adverse events were associated with the AUCt and AUCu values measured on day 1. As shown in Fig. 5, no significant associations were observed between the PFS values and AUCt and AUCu values of regorafenib, M2, and M5.

## Discussion

To the best of our knowledge, we demonstrated for the first time that regorafenib and its active metabolites, M2 and M5, inhibit MICA shedding in human CRC cell lines (Fig. 2A) without cytotoxic effects. The potencies of M2 and M5 to inhibit sMICA release were higher than that of parent regorafenib (Fig. 2B). Rothe et al. designed an “immunoligand” that binds to both colon carcinoma antigen and a cytotoxicity receptor on NK cells. The immunoligand is similar to mMICA expressed on the surface of CRC cells and resulted in potent anti-tumor activity in vitro and in vivo by activating innate as well as adaptive immune cells, including NK cells [24]. By contrast, sMICA acts as a decoy to prevent anti-cancer surveillance by NK cells [25]. Therefore, a decrease in sMICA levels is considered to improve clinical outcomes in patients with cancer by activating NK cell-mediated cytotoxicity. Our present results (Fig. 2) suggest that NK cell activation could potentially occur to a greater extent after regorafenib treatment, for which the anti-tumor mechanisms related to immunology are currently unknown.

Our in vitro data prompted us to evaluate the effects of regorafenib and its active metabolites, M2 and M5, on plasma sMICA levels and the associations between plasma sMICA levels and the clinical efficacy of regorafenib in patients with CRC. As was expected, responders showed significantly lower sMICA levels just before the start of regorafenib treatment than non-responders (Fig. 3A). Furthermore, plasma sMICA levels were significantly negatively associated with PFS and OS, and the Kaplan–Meier analysis showed a significant difference in PFS between the low and high plasma sMICA groups (Fig. 4B and C). These results appear to be consistent with those of a previous report on HCC patients with chronic hepatitis, wherein high levels of sMICA in plasma were shown to be an indicator of poor prognosis, most likely because sMICA acts as immunological decoy to decrease NK cell-medicated cytotoxicity [8].

In a previous prospective clinical study on patients with CRC who received regorafenib [23], patients with higher AUCu values of M2 or M5 on day 1 showed significantly shorter PFS than others. This was likely due, at least in part, to treatment discontinuation as a result of adverse events, especially events that occurred during first cycle. In the present study, we only evaluated the effects of sMICA levels on survival benefit of regorafenib in patients who ceased treatment due to tumor progression and excluded patients who discontinued treatment because of drug-related adverse events. No significant associations were observed between the PFS values and the AUCt and AUCu values of regorafenib, M2, and M5 (Fig. 5), suggesting a minimal contribution of the pharmacokinetics of regorafenib and its active metabolites to the interindividual variability in PFS, but large contribution of pharmacokinetics to interindividual variability in regorafenib-induced adverse events.

Regorafenib and its active metabolites inhibited MICA shedding in human CRC cells (Fig. 2A, 2B). We clarified that ADAM10 and ADAM17 partially contributed to MICA shedding among various ADAMs and MMPs that were reported to enzymatically cleave mMICA to release sMICA in humans [26]. However, the mechanism by which regorafenib inhibits MICA shedding in CRC cells remains unclear. In HCC cells, MICA shedding was shown to be mediated by ADAM9, ADAM10, and ADAM17 [7], wherein regorafenib inhibits MICA shedding by decreasing mRNA levels of ADAM9 and ADAM10 [16]. However, regorafenib, M2, and M5 did not target these ADAMs in CRC (data not shown). Further studies are warranted to elucidate the mechanism of MICA shedding inhibition induced by regorafenib or its active metabolites in CRC cells.

The present study has several limitations. First, the sample size was relatively small because patients who quit the treatment due to adverse events were excluded from evaluation of whether sMICA levels could be used as a biomarker of the therapeutic efficacy of regorafenib. We did not find significant associations between sMICA levels and OS in a Kaplan–Meier analysis, partly due to such a small clinical sample size. Second, we could not clarify the entire mechanism by which regorafenib and its active metabolites inhibit MICA shedding in CRC cells. Comprehensive analyses using multi-omics technologies are underway to identify genes and molecules responsible for the inhibition of MICA shedding, expectedly deciphering novel molecular immunological modes of action.

## Conclusions

This study clarified that treatment of CRC cells with regorafenib or its active metabolites M2 and M5 for 48 h significantly decreased sMICA levels, presumably due to the inhibition of MICA shedding. Patients who showed long median PFS had significantly lower sMICA levels than those with shorter median PFS. Importantly, manipulating MICA is an attractive strategy for the immunological control of CRC. In this small cohort, baseline sMICA levels could be the predictive biomarker for the efficacy of regorafenib.

Further analysis is required to clarify whether manipulating MICA will lead to better outcome.

## Data Availability

All data generated or analyzed during this study are included in the manuscript.

## Abbreviations

CRC: Colorectal Cancer
MICA: MHC Class I polypeptide-related sequence A
sMICA: Soluble MICA
NK: Natural Killer
mMICA: Membrane-bound MICA
HCC: Hepatocellular Carcinoma
ADAMs: A Disintegrin and Metalloproteases
PFS: Progression-Free Survival
CCK8: Cell Counting Kit-8
ELISA: Enzyme Linked Immunosorbent Assay
qRT-PCR: Quantitative Reverse Transcription-polymerase Chain Reaction
AUCu: Area Under the unbound plasma Concentration–time Curve
AUC: Area Under the Curve
ROC: Receiver-Operating Characteristics
OS: Overall Survival
AUCt: Total plasma concentration base AUC
